# Purpura Fulminans Secondary to *Streptococcus pneumoniae* Meningitis

**DOI:** 10.1155/2012/508503

**Published:** 2012-01-26

**Authors:** Erick F. Alvarez, Karen E. Olarte, Mayur S. Ramesh

**Affiliations:** ^1^Infectious Diseases Division, Henry Ford Hospital, 2799 W. Grand Blvd., Detroit, MI 48202, USA; ^2^Internal Medicine Residency Program, Henry Ford Hospital, Detroit, MI 48202, USA

## Abstract

Purpura fulminans (PF) is a rare skin disorder with extensive areas of blueblack hemorrhagic necrosis. Patients manifest typical laboratory signs of disseminated intravascular coagulation (DIC). Our case describes a 37-year-old previously healthy man who presented with 3 days of generalized malaise, headache, vomiting, photophobia, and an ecchymotic skin rash. Initial laboratory workup revealed DIC without obvious infectious trigger including unremarkable cerebrospinal fluid (CSF) biochemical analysis. There was further progression of the skin ecchymosis and multiorgan damage consistent with PF. Final CSF cultures revealed *Streptococcus pneumoniae*. Despite normal initial CSF biochemical analysis, bacterial meningitis should always be considered in patients with otherwise unexplained DIC as this may be an early manifestation of infection. PF is a clinical diagnosis that requires early recognition and prompt empirical treatment, especially, in patients with progressive altered mental status, ecchymotic skin rash, and DIC.

## 1. Introduction

Purpura fulminans (PF) is an unusual skin manifestation of disseminated intravascular coagulation (DIC) associated with infection and/or sepsis. It is characterized by tissue necrosis, small vessel thrombosis in the setting of DIC. PF often leads to end organ damage with resultant profound morbidity and mortality. We describe a case of PF secondary to *Streptococcus pneumoniae* infection.

## 2. Case Presentation

**Figure 1:**
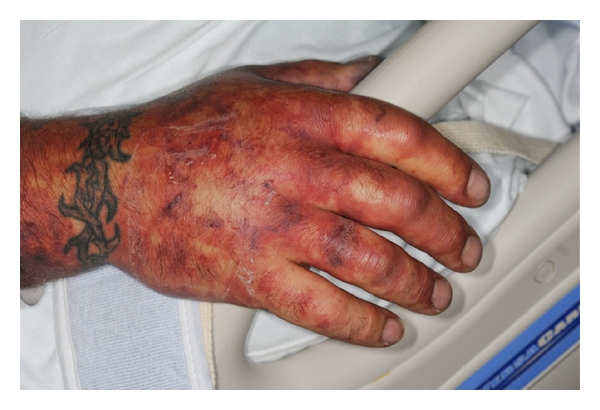
Ecchymotic nonblanching macular lesions on the hand.

**Figure 2:**
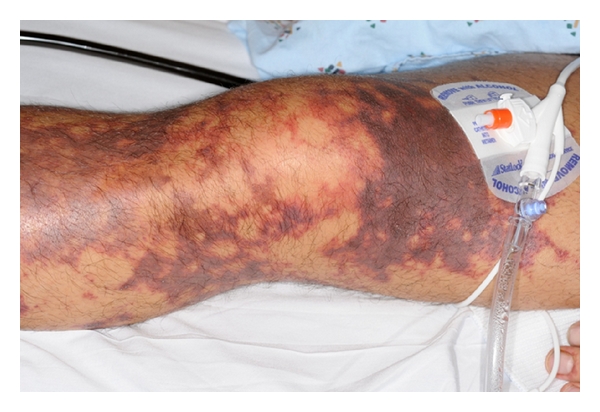
Ecchymotic nonblanching macular lesions on the lower extremity.

**Figure 3:**
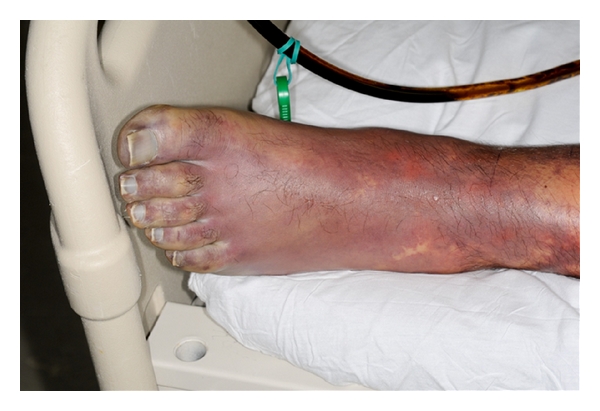
Ecchymotic nonblanching macular lesions on the foot.

A 37-year-old previously healthy man presented to the emergency department with 3 days of generalized malaise, headache, nausea, vomiting, photophobia, and an ecchymotic skin rash. Admission physical evaluation revealed that he was tachycardic, somnolent, but oriented without nuchal rigidity or focal neurologic signs. He had a diffuse ecchymotic nonblanching macular rash on his extremities and abdomen (Figures 1, 2, and 3).

Initial laboratory tests revealed leukocytosis (25 × 10^9^/L), thrombocytopenia (32 × 10^9^/L), and normal hemoglobin. Coagulation studies showed prothrombin time of 15.6 seconds, partial thromboplastin time of 37 seconds, fibrinogen 10.9 *μ*mol/L, and D-dimer >20000 *μ*g/L consistent with DIC. Liver and renal function tests revealed AST 117 U/L, ALT 65 U/L, total bilirubin 20.2 *μ*mol/L, BUN 20.3 mmol/L, and creatinine 247.52 *μ*mol/L. Initial cerebrospinal fluid (CSF) analysis was within normal limits (WBC 1 × 10^6^/L, RBC 1 × 10^6^/L, glucose 2.9 mmol/L, protein 0.47 g/L, and no organisms seen on gram stain). Serology for HIV, EBV, CMV, and hepatitis viruses was negative, as well as serologic testing for *rickettsia*. Other tests revealed serum lactate 2 mmol/L, CPK 512 U/L, and LDH 1008 U/L. Antinuclear antibody and rheumatoid factor were negative with normal serum complement levels. Admission chest radiography and a computerized tomography of the head were normal. A peripheral smear showed thrombocytopenia with low-grade microangiopathic hemolytic anemia (MAHA).

He was started on intravenous (IV) empiric antibiotics including vancomycin, cefepime, and metronidazole for a clinical suspicion of sepsis and also received platelet transfusion and IV heparin for DIC. Multiple blood cultures done during the hospitalization did not grow any organisms. Two days into admission, CSF cultures grew *Streptococcus pneumonia*, and antibiotics were switched to IV ceftriaxone (2 grams every 12 hours). His skin lesions progressed rapidly to hemorrhagic bullae. Skin biopsy showed widespread hemorrhage with focal thrombosis. The clinical picture of rapidly progressive ecchymotic skin rash in our patient with DIC secondary to *Streptococcus pneumoniae *infection was consistent with a diagnosis of PF, and the skin biopsy confirmed the same.

The patient's hospital course was complicated by a transient worsening of his mental status, distal extremities thrombosis, and worsening renal function that required hemodialysis. With support care and antibiotic therapy (for a total of two weeks), he improved clinically. Upon discharge patient returned to his baseline mental status, his skin lesions cleared except the lesions on his lower extremities, and he remained on hemodialysis.

## 3. Discussion

Purpura fulminans is a rare, severe skin disorder associated with DIC that primarily affects children and infants. Extensive areas of skin develop blueblack hemorrhagic necrosis; biopsy reveals small-vessel microthrombi and occasionally mild vasculitis. In our patient the clinical findings are suggestive of microangiopathic thrombosis with hemolysis secondary to DIC and purpura fulminans. The pathogenesis is unknown, but histologic findings have been likened to the animal model of consumptive coagulopathy [1]. It has also been suggested that the development of acquired defects in the protein C pathway similar to two other protein C deficiency states, namely, neonatal purpura fulminans and warfarin-induced skin necrosis [2, 3]. In our patient, protein C was 87% (normal), and protein S was 15% (low).

The mortality rate has recently been significantly reduced in purpura fulminans, largely because of more widespread use of therapeutic heparinization in these patients and aggressive replacement of platelets and coagulation factors [4, 5]. Our patient also improved clinically with these interventions.

The most common organisms producing DIC are bacterial, especially the gram-negative bacteria (*meningococci*, *Haemophilus influenzae*, *Aerobacter*, and others) but also gram-positive organisms (*Staphylococcus aureus*, group B streptococci, *Streptococcus pneumonia*, and *Bacillus anthracis*). DIC is also associated with disseminated viral (varicella, measles, and rubella), rickettsial (Rocky Mountain spotted fever), fungal, mycoplasma, and parasitic infections. In our patient, *Streptococcus pneumoniae* was eventually grown from the CSF.

Clinically, patients with PF present with painful, erythematous macular lesions, and ecchymoses. These lesions evolve into painful indurated, well-demarcated purple papules with erythematous borders. Finally, they progress to necrosis with the formation of bullae and vesicles. In our patient, these skin lesions helped in the early diagnosis and eventual successful treatment of PF.

*Streptococcus pneumoniae* is the most common cause of meningitis in adults, particularly in elderly [6, 7]. It is common to have completely normal CSF cellularity and biochemistry in patients with bacterial meningitis [8, 9]. Despite these typical CSF findings, the spectrum of CSF values in bacterial meningitis is so wide that the absence of one or more of the typical findings is of little value [8, 9]. Similar to our patient where no abnormalities were found in the initial CSF analysis, in a series of 696 episodes of community-acquired bacterial meningitis, 12 percent had none of the characteristic CSF findings [9]. The explanation for minimal CSF abnormalities cannot usually be identified. Potential causes include early presentation, recent prior antibiotic therapy, and neutropenia. The normal CSF seen initially in our patient may be due to his early presentation to the ED.

Bacterial meningitis should be kept in the differential diagnoses in patients with otherwise unexplained DIC, especially with progressive altered mental status despite initial normal CSF analysis. Early clinical recognition of PF is essential for the successful outcome.
